# Molecular identification of triticale introgression lines carrying leaf rust resistance genes transferred from *Aegilops kotschyi* Boiss. and *Ae. tauschii* Coss

**DOI:** 10.1007/s13353-021-00635-2

**Published:** 2021-05-14

**Authors:** Michał T. Kwiatek, Jolanta Belter, Waldemar Ulaszewski, Roksana Skowrońska, Aleksandra Noweiska, Halina Wiśniewska

**Affiliations:** 1 Department of Genomics, Institute of Plant Genetics of the Polish Academy of Sciences, Strzeszyńska 34, 60-479 Poznań, Poland; 2 Department of Genetics and Plant Breeding, Poznan University of Life Sciences, Dojazd 11, 60-632 Poznań, Poland

**Keywords:** *Aegilops*, Genomic in situ hybridization. Leaf rust, Molecular markers, Resistance genes, Triticale

## Abstract

Triticale (× *Triticosecale* Wittmack) is a commercial hybrid harboring wheat (*Triticum* sp.) and rye (*Secale cereale* L.) genomes. The limited genetic diversity of this crop resulted in the collapse of fungal disease resistance. Leaf rust disease, caused by *Puccinia triticina* Eriks., is reported to reduce the triticale yield significantly (more than 30%). There is a need to enlarge the genetic variability of this crop including leaf resistance genes. The main aim of this research was to evaluate the leaf rust resistance of the offspring of translocation lines of triticale carrying chromatin of *Ae. tauschii* and *Ae. kotschyi*. A reaction of seedlings of 200 plants of two triticale-*Aegilops* translocation lines (Bogo-2D^t^.2R and Sekundo-2S^k^.2R) was compared after inoculation with a natural mixture of *P. triticina* races, specific to triticale in controlled condition. Before inoculation, each plant was screened using molecular cytogenetics and molecular markers linked to leaf rust resistance genes. The presence of *Aegilops* chromosome segments was confirmed using genomic in situ hybridization (GISH). *Lr39* and *Lr54* leaf rust resistance genes were identified using *Xgdm35* and *S14* molecular markers, respectively. After inoculation, a significant improvement of resistance severity was observed in Sekundo-2S^k^.2R in comparison with triticale cv. Sekundo plants. The resistance level of Bogo-2D^t^.2R did not differ compared with triticale cv. Bogo plants. It was shown that *Lr39* gene did not increase the leaf rust resistance level of triticale cv. Bogo.

**Supplementary Information:**

The online version contains supplementary material available at 10.1007/s13353-021-00635-2.

## Introduction

Triticale (× *Triticosecale* Wittmack 2n = 6x = 42 chromosomes, AABBRR) is a hybrid crop composed of wheat (*Triticum* sp.) and rye (*Secale cereale* L.) genomes, which is commercially used for forage, food, and biofuel production (Meale and Mcallister 2015). Leaf rust, caused by fungus *Puccinia triticina* Eriks, is one of the most destructive foliar diseases of triticale worldwide. This disease occurs mainly on the leaf blade, producing small elliptical orange-red pustules on the upper surface and causing premature defoliation. It causes both yield losses and downgrading in quality (Hanzalová and Bartoš 2011) and its natural populations virulence is higher on triticale in comparison to wheat (Mikhailova et al. 2009). During the evolution, plants have elaborated large number of resistant genes (R genes) as a part of their defense system. R genes encode receptors, recognizing, produced by pathogen, avirulence gene-dependent elicitors (De Wit 1997). In turn, triticale suffers lack of the evolution process. The genetic pool of wheat and rye forms for cross-hybridizations were relatively narrow and it had and significant effect on low genetic variability of this crop (Kwiatek and Nawracała 2018). This is the most possible reason of the resistance collapse of triticale in last two decades (Arseniuk and Góral 2015).

The most economical and environmentally friendly approach to reduce yield losses, caused by leaf rust diseases, is the host plant genetic resistance. This kind of resistance is crucial for farming with no use or limited pesticides. Cultivars of wheat with improved disease resistance have been successfully developed, using *Lr* genes, in breeding programs. Proteins encoded by some *Lr* genes have evolutionary conserved DNA motifs such as nucleotide binding site (NBS) and leucine repeat rich (LRR) (Kolmer 2013). More than 80 genes for resistance to leaf rust have already been catalogued on wheat and its relatives (McIntosh et al. 2019).

The *Aegilops* genus is the closest wild relative of *Triticum* which includes cultivated forms of wheat. Several *Aegilops* accessions have resistance to fungal pathogens of cereals. New gene variants derived from *Aegilops* species, which are related to biotic stress resistance are considered as sources for improving the stress tolerance of wheat and triticale (Schneider et al. 2008; Kwiatek and Nawracała 2018). What is more, the polyploid nature of the wheat or triticale genome facilitates the survival of genetically unbalanced genomic material within the nucleus. This enables the introduction of alien DNA into the wheat/triticale genome since even the addition of whole chromosome arms from a different genome may be tolerated (Kwiatek and Nawracała 2018).

The first alien *Lr* gene that has ever been introduced into wheat genome was *Lr9*. This gene was transferred by Sears (1956) from *Ae. umbellulata* into hexaploid wheat through X irradiation induced translocation. A number of other leaf rust resistance genes were transferred from *Aegilops* species and commercially utilized in wheat (Schneider et al. 2008). *Aegilops tauschii* Coss, the D-genome donor of wheat, has been a rich source of leaf rust resistance genes (Rayburn and Gill 1987). Several leaf rust resistance genes (*Lr22a* (2D), *Lr32* (3D), *Lr39* (2D) have been transferred into triticale from *Ae. tauschii* (Kwiatek et al., 2015; Majka et al. 2018).

Homoeologs pairing and recombination have been widely used to transfer valuable resistance genes from alien chromosome to wheat chromosome. Chromosome translocations of leaf rust resistance gene are said to occur spontaneously when wheat-*Aegilops* introgression lines are backcrossed (Faris et al. 2002). One of the most efficient techniques of introducing alien chromatin into wheat or triticale is recombination-based chromosome engineering (Kwiatek and Nawracała 2018). Marais et al. (2005) have introduced, *Ae. kotschyi* derived, leaf and stripe rust-resistant genes *Lr54* and *Yr37*, respectively, to the genome of wheat. Double monosomic for 2D chromosome of wheat and a 2S^k^ chromosome of *Ae. kotschyi* were used in this study. A translocation that was formed following centric breaking and subsequent fusion of an *Ae. kotschyi* chromosome 2S^k^L arm with 2DS arm of wheat. Ulaszewski et al. (2019) produced Robertsonian translocations (RobTs) in the progeny of triticale cv. Sekundo plants with monosomic substitution of *Ae. kotschyi* chromosome 2S^k^(2R). 2S^k^.2R compensatory RobTs were produced using utilized ditelosomic lines of triticale carrying 2RS (short arm) and 2RL (long arm) telosomic chromosomes. The authors reported that six plants carried T2RS.2S^k^L translocation. Moreover, Kwiatek (2018) used the same strategy and developed five plants carrying 2D^t^.2R compensatory RobTs (introduced from *Ae. tauschii* into cv. Bogo).

Both *Lr39* and *Lr54* resistance genes were reported to be effective against leaf rust (Raupp et al. 2001; Marais et al. 2005, respectively) in *Aegilops*–wheat translocation lines. Here we present the initial studies on the possibilities to enhance the triticale leaf rust resistance using *Aegilops*–triticale translocation lines. The aim of this work was to evaluate the resistance of the offspring of translocation lines of triticale with chromatin of *Ae. tauschii* and *Ae. kotschyi* at seedling stage for infection of natural mixture of *Puccinia triticina* Eriks in controlled condition.

## Materials and methods

### Plant material

**Fig. 1:**
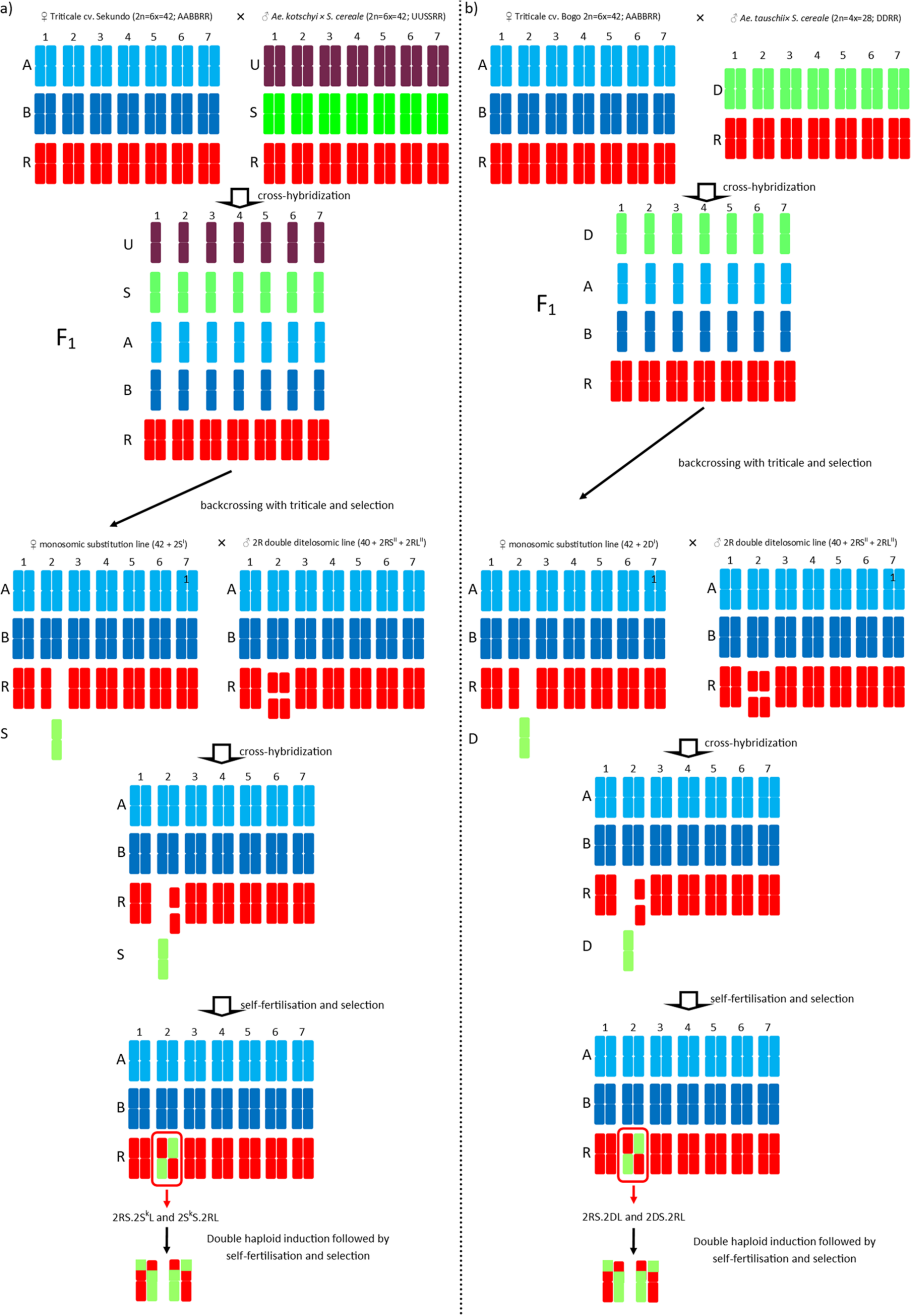
Origination of **(a**) Sekundo-2S^k^.2R translocation line and (**b**) Bogo-2D^t^.2R translocation line

An offspring of two triticale lines carrying two compensated chromosome translocations (2D^t^.2R and 2S^k^.2R) (Table 1). Alien chromatin segments were introduced into triticale cultivars: Bogo and Sekundo from *Ae. tauschii* and *Ae. kotschyi*, respectively (Kwiatek et al. 2018; Ulaszewski et al. 2019) (Fig. 1). Offspring plants of triticale translocation lines were used for marker-assisted selection of plants carrying *Lr39* and *Lr54* leaf rust resistance genes. Selected plants were called Bogo-2D^t^.2R and Sekundo-2S^k^.2R and evaluated for leaf rust symptoms. Additionally, each experiment involved two negative controls. First control included 20 plants of each of two triticale cultivars (Bogo and Sekundo), which were used in order to compare the infection response of translocation plants. The second control involved 30 plants of bread wheat cv. Michigan Amber, which is reported to be highly susceptible to leaf rust infection (Kolmer et al. 2013). Moreover, 30 plants of KS90WGRC10 wheat line, which was reported to carry *Lr39* loci derived from *Ae. tauschii* TA1675 (Raupp et al. 2001), were used as positive control to check the virulence of a natural mixture of leaf rust urediniospores on *Lr39* gene.

**Table 1:** Cytogenetic characterization of the introgression line of triticale carrying chromatin fragments with leaf rust resistance genes *Lr39* and *Lr54* transferred from *Aegilops kotschyi* and *Ae tauschii* into triticale cv. Bogo and Sekundo, respectively

Translocation lines of triticale (type of chromosome translocation)	Number of offspring plants	Transferred leaf rust resistance genes (chromosome localization)	Donor of alien chromatin	Number of plants offspring carrying	
				Chromosome translocations	Chromosome substitution
Bogo-2D^t^.2R (RobTs 2D^t^.2R)	100	*Lr39* (2D^t^)	*Ae. tauschii*	100 (2DS.2RS-2RL and 2RS.2DS-2DL)	0
Sekundo-2S^k^.2R (RobTs 2S^k^.2R)	100	*Lr54* (2S^k^)	*Ae. kotschyi*	18 (2S^k^S.2RS-2RL and 2RS.2S^k^S-2S^k^L)	82 (2S^k^/2R)

### SSR marker screening

Genomic DNA of two *Aegilops* accessions, two donor triticale cultivars and 200 plants with alien chromatin introgression (2 combinations × 100 plants) were isolated using Plant DNA Purification Kit (EurX Ltd., Gdańsk, Poland). All primers (Table 2) were manufactured by Sigma-Aldrich (Merck). PCR reactions were performed in a LabCycler thermal cycler (SensoQuest Biomedizinische Elektronik, Goettingen, Germany). The 20-µL PCR reaction consisted of 150-nM each primer, 0.2 mM of each nucleotide, 1.5-mM MgCl_2_, 0.2 units of Taq-DNA hot-start polymerase (TaqNovaHS, Blirt, Poland), and 50 ng of genomic DNA as a template. A typical PCR procedure was as follows: 5 min at 95 °C, then 35 cycles of 30 s at 94 °C, 30 s at 55 or 60 °C (depending on the primer, Table 2), 1 min at 72 °C, and 5 min at 72 °C. 0.5µL Midori Green Direct (Nippon Genetics Europe) was added to each amplification product, ran on 2% agarose gel (Sigma), and then visualized and documented using EZ GelDoc System (BioRad). Each sample was tested twice. Third, additional run was made in the case of discrepancy in the results.

**Table 2:** Primer sequences and PCR conditions used for markers identification of *Lr39* and *Lr54* genes

Molecular marker	Leaf resistance gene	Primer sequences (5′ to3′)	Amplification temperature (°C)	Fragment size (bp) in Aegilops	Fragment size in triticale	Source
*Xgdm35*	*Lr39*	CCTGCTCTGCCCTAGATACG	55 °C	190	null	Pestsova et al. 2000
		ATGTGAATGTGATGCATGCA				
*S14—297*	*Lr54*	CATGCAGAAAACGACACACC	60 °C	410	null	Smit 2013
		GGTAAGTGGTCAGGCGTTGT				

### *Genomic *in situ* hybridization*

Chromosome spreads of 200 plants were prepared using enzymatic digestion and squashing protocol described by Kwiatek et al. (Kwiatek et al., 2017). Molecular probes for alien chromatin identification were prepared using total genomic DNA of *Ae. sharonensis* and *Ae tauschii*, which were purified using GeneMATRIX Plant and Fungi DNA Purification Kit (EURx, Gdansk, Poland). *Ae. sharonensis* is reported as a donor of S^k^-genome of *Ae. kotschyi* (Ruban and Badaeva 2018). Genomic DNA of *Aegilops* species was labeled by nick translation (NickTranslation Kit, Merck) with digoxigenin-11-dUTP dye (Merck). DNA of rye [(*Secale cereale* L.), cv. Imperial, USDA, Aberdeen, Idaho, USA] was labeled in the same manner (nick translation) using tetramethyl-rhodamine-5dUTP in order to detect R-genome chromatin. Blocking DNA from *T. durum* Desf. (2*n* = 4*x* = 28 chromosomes; AABB; cv. Ceres; HR Smolice; Poland) was used to detect A- and B-genome chromosomes of triticale. Blocking DNA was sheared by boiling for 30–45 min and used at a ratio of 1:50 (probe/block). Genomic in situ hybridization (GISH) was carried out according to previously published protocols (Kwiatek et al. 2016). The reactions were followed by post-hybridization washes in 0.1 × SSC (Saline Sodium Citrate, Merck) buffer at 42 °C (3 washes, for 5 min each; stringency: 73%, according to Schwarzacher and Heslop-Harrison 2000) to dissociated imperfect matches, which provided only specifically bound probe on target sequences. Chromosome spreads were examined with the Olympus BX 61 automatic epifluorescence microscope equipped with Olympus XM10 CCD camera. Olympus Cell-F (version 3.1; Olympus Soft Imaging Solutions GmbH: Münster, Düsseldorf, Germany) imaging software and PaintShop Pro X5 software (version 15.0.0.183; Corel Corporation, Ottawa, ON, Canada) were used for image processing and documentation.

### Evaluation of leaf rust symptoms in growth chamber

Evaluation of leaf rust was carried out in in growth chamber (at IPG PAS) using a natural mixture of leaf rust urediniospores, which were collected from triticale fields in three localizations in Wielkopolska region: IPG PAS Experimental Station in Cerekwica, Poland (52°31′16″N 16°41′30″E); Experimental Station of the Poznan University of Life Sciences (PULS), Dłoń, Poland (51°41′22″N 17°04′23″E); and Experimental Garden of the Department of Genetics and Plant Breeding (PULS) in Poznań (52°25′26″N 16°54′07″E). Plants at three-leaf stage were challenged with leaf rust by spraying urediniospore solution containing 0.1% Tween 20. The inoculated plants were then incubated in a humid growth chamber free from light for 15 days. After inoculation, the plants were maintained under a day/night photoperiod of 18/6 h, a temperature of 16–22 °C. Winter triticale cv. Bogo and Sekundo, winter wheat cv. Michigan Amber were taken as the susceptible controls. KS90WGRC10 wheat line was used as a positive control for *Lr39* gene. The infection type of each individual was scored at three timepoints (5, 10 and 15 days post inoculation—*dpi*) using an infection scale adapted from (Roelfs 1988) and transformed into nine-grade scale (1, high resistance; 9, susceptibility, Table 2; McNeal et al. 1971). The means of scores of leaf rust symptoms were compared between translocation lines and controls including acceptor cultivars of triticale and Michigan Amber wheat using analysis of variance (ANOVA) and Tukey’s highest significant difference (HSD) test (Supporting Information 1, 2, and 3).

## Results

### Marker-assisted selection

**Fig. 2:**
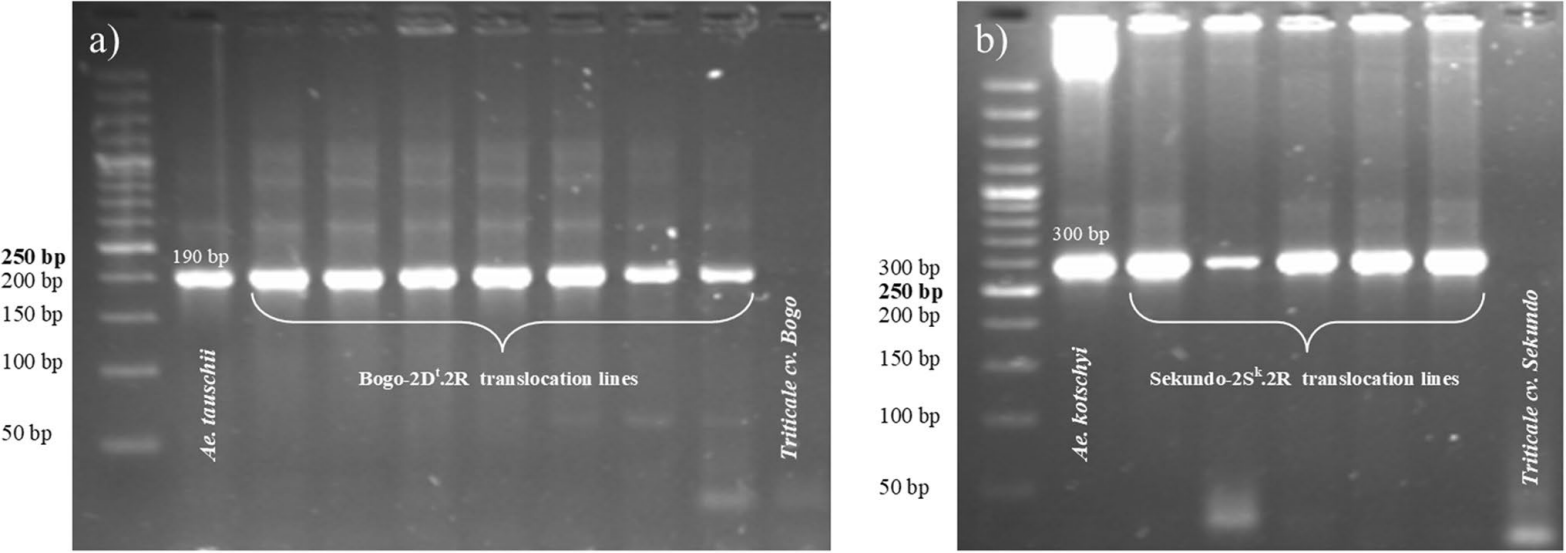
Amplification products of (**a**) *Xgdm35* marker linked to *Lr39* leaf rust resistance gene and (**b**) *S14* marker linked to *Lr54* leaf rust resistance gene

In the first step, marker-assisted selection was used to choose two hundred plants from the progeny of each of two *Aegilops*-triticale translocation lines carrying *Lr39* or *Lr54* genes. Each sample was tested twice. One hundred offspring plants of 2D^t^.2R triticale translocation line (an introgression of *Ae. tauschii* chromatin) showed a 190 bp product afer PCR reaction with *Xgdm35* marker linked to *Lr39* resistance gene (Fig. 2a). The same product was observed for *Ae. tauschii* control. No amplification product was observed for triticale cv. “Bogo.” 100 plants with *Lr54* gene loci were selected from the recombinants derived from 2S^k^.2R triticale translocation line (an introgression of *Ae. kotschyi* chromatin) by the use of *S14* marker. A 300 bp amplification product was observed for *Ae. kotschyi* control and hybrid plants (Fig. 2b). Lack of amplification products was characteristic for triticale cv. “Sekundo.” Both groups of plants were evaluated for the evaluation of infection in the further steps of the experiment.

### *Genomic *in situ* hybridization*

**Fig. 3:**
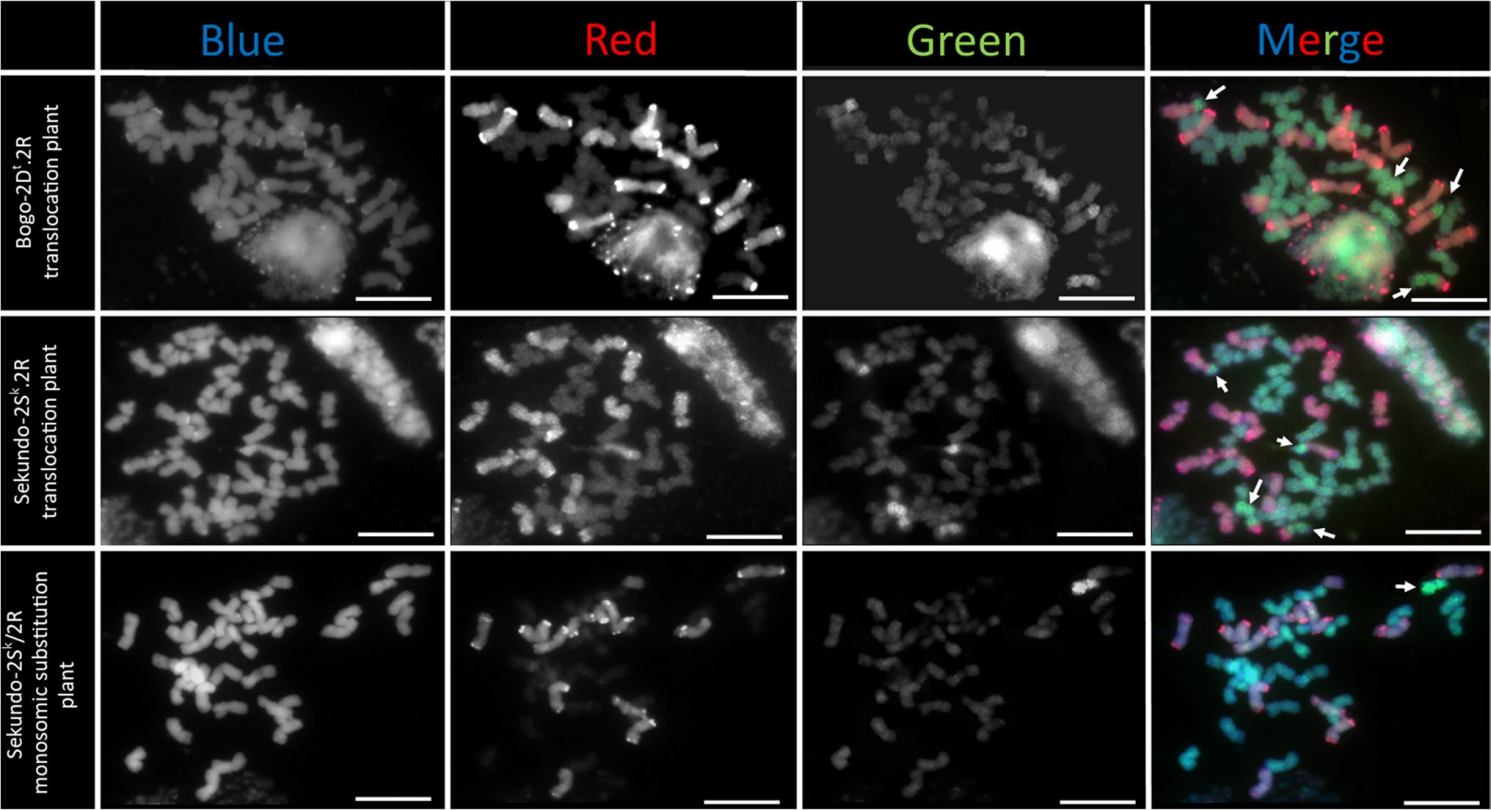
Karyotypes of: Bogo-2D^t^.2R translocation plant; Sekundo-2S^k^.2R translocation plant and Sekundo-2S^k^/2R monosomic substitution plant examined by genomic in situ hybridization. Total genomic DNA of *Aegilops* sp*.* (green channel) and rye (R-genome chromatin; red channel) were used as probes. Total genomic DNA of *Triticum durum* (A- and B-genome chromatin; blue channel) was used as a blocker. Three channels were combined to merge image. Arrows indicate *Aegilops* chromatin. Scale bar: 10 µm

In total, 200 selected plants were used for genomic in situ hybridization experiment. This approach aimed in the evaluation of the amount of alien chromatin segments in triticale genetic background (Table 1). Within the first group (100 plants), two types of chromosome translocations including large segment of 2D^t^ chromosome with the centromere region, and short distal chromosome translocations were observed (Fig. 3). The chromosome sets of 100 plants belonging to second group were more diversified. Eighteen plants showed a two different types of chromosome translocations, including large parts of 2S^k^ chromosome with the centromere region or short segments of this chromosome located distally in the subtelomeric region of 2R chromosomes (Fig. 3). Eighty-two plants showed complete 2S^k^ chromosomes (Fig. 3).

### Evaluation of leaf rust symptoms

**Fig. 4:**
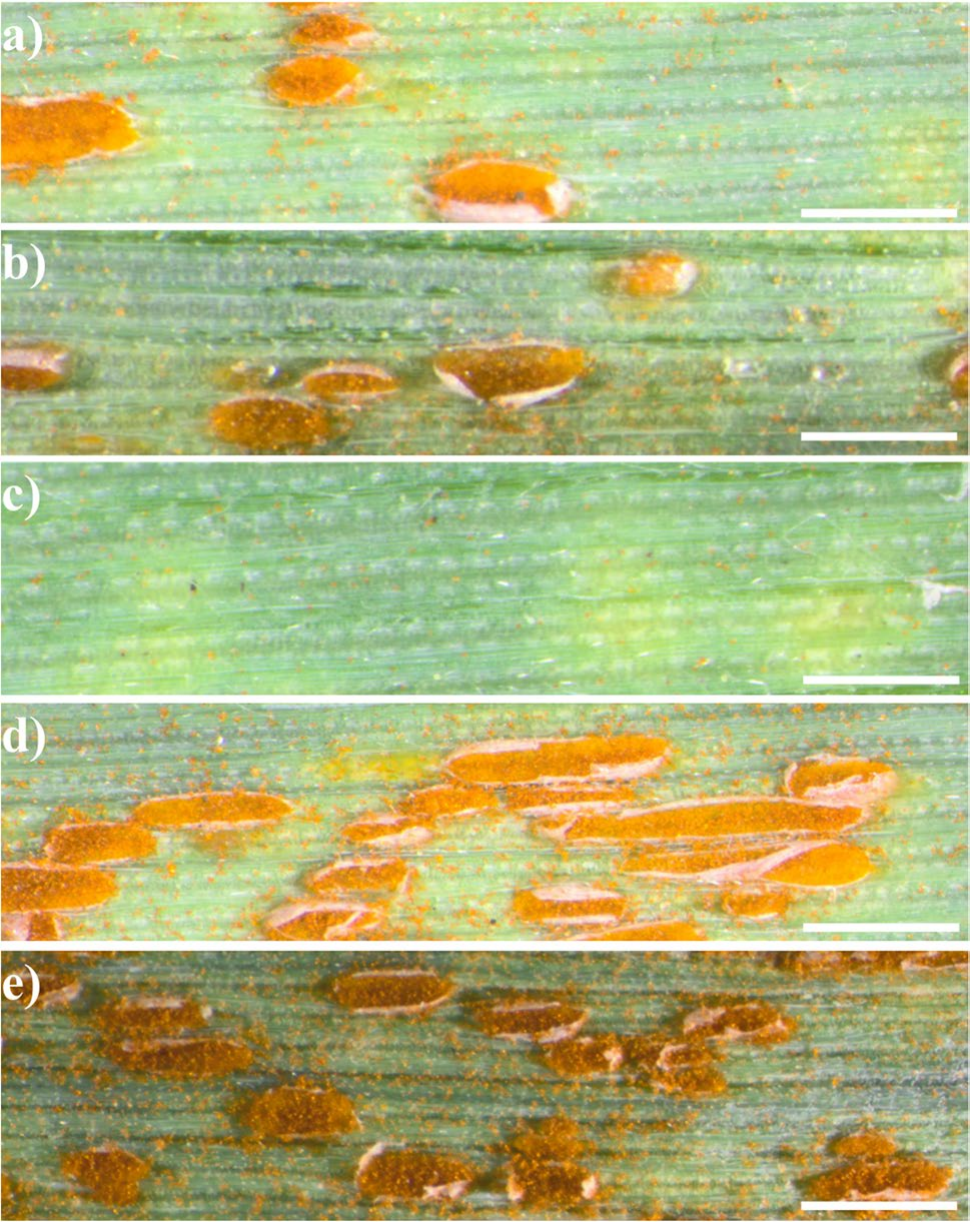
Symptoms of leaf rust infection on the leaves of (**a**) Bogo-2D^t^.2R translocation plant; (**b**) triticale cv. Bogo; (**c**) Sekundo-2S^k^.2R translocation plant (**d**) triticale cv. Sekundo, and (**e**) Michigan Amber (wheat, positive control). Scale bar: 1 mm

The phenotypes of 200 plants belonging to two combinations (100 plants each) of triticale translocation lines (Bogo-2D^t^.2R and Sekundo-2S^k^.2R) were evaluated at the seedling stage in the growth chamber and compared to phenotypes of acceptor cultivars of triticale (Bogo and Sekundo, respectively). Thirty plants of Michigan Amber wheat and 30 plants KS90WGRC10 wheat line were used as additional controls (Table 3). The mean score for Michigan Amber plants (8.8) showed that the inoculation solution was effective for induction of the infection (Fig. 4). The comparison of the means of infection levels, which were calculated for triticale cultivars (donors) showed that cv. Bogo represented a higher resistance level (4.07) compared with cv. Sekundo (7.17 at HSD_0.01_ = 0.43) (Fig. 4; Table 3; Supporting Information 1). The mean score of three independent evaluations of infection level (5, 10 and 15 dpi) in Bogo-2D^t^.2R plants varied between 3.94 and 4.2 (Table 3, Supporting Information 2). The results were comparable with the mean sore of infection of triticale cv. Bogo (4.07) and KS90WGRC10 (4.13), which is reported to carry *Lr39* gene (Table 3; Supporting Information 2). The second group of Sekundo-2S^k^.2R plants revealed seedling resistance. The infection rates were 1.59, 1.65, and 1.67 (5, 10 and 15 dpi) (Table 3; Supporting Information 3) and did not differ significantly considering the evaluation timepoints (Supplementary Information 3). In comparison, plants of cv. Sekundo showed limited resistance (7.17). The Tukey HSD test revealed that the differences in infection scores between Sekundo-2S^k^.2R and cv. Sekundo plants were significant at α = 0.01 level (HSD_0.01_ = 0.31) (Supporting Information 3).

**Table 3:** Means of infection levels scored 5, 10 and 15 days post inoculation (dpi)

Plant material	Number of plants tested	Means of infection levels			
		5 dpi	10 dpi	15 dpi	mean
Bogo-Lr39 (introgression line)	100	3.94	4.13	4.2	4.09
Triticale cv. Bogo (donor control)	30	4.0	4.1	4.1	4.07
KS90WGRC10 (wheat control; *Lr39*)	30	3.9	4.2	4.3	4.13
Sekudo-Lr54 (introgression line)	100	1.59	1.65	1.67	1.64
Triticale cv. Sekundo (donor control)	30	7.0	7.1	7.4	7.17
Michigan Amber (wheat control)	30	8.6	8.9	8.9	8.8

## Discussion

The main aim of this research was to evaluate the effectiveness of *Lr39* and *Lr54* leaf rust resistance genes, which were transferred separately into two triticale cultivars (Bogo and Sekundo) through development of the 2*Ae*.2R compensating chromosome translocation lines. Genomic in situ hybridization supported by the screening of molecular markers linked to leaf rust resistance genes allowed to select plants for inoculation tests. Cytogenetic analyses showed that 2D^t^.2R Robertsonian translocations (RobTs) have been rearranged. All 100 Bogo-2D^t^.2R plants showed different locations of chromosome breakpoints (Fig. 3) compared with compensating Robertsonian translocations, which were reported in the parental forms (Ulaszewski et al. 2019; Kwiatek et al. 2018). Induced reduction of introgressed whole arms of alien chromosomes was reported multiple times (Howell et al. 2014; Lukaszewski 2000; 2010). In these studies 1RS.1BL and 1BS.1RL Robertsonian translocations were produced by centric misdivision of univalents. In our study plants with RobTs were self-pollinated. It could be possible that 2D^t^S.2R and 2RS.2D^t^L chromosomes paired and recombined and novel configurations of those two chromosomes appeared in the offspring (Fig. 3). Considering the homoeology along 2D^t^S.2R and 2RS.2D^t^L chromosomes, only centromere regions might be supposed to be responsible for pairing during meiosis. It was reported that centromere structure of 1BS.1RL centric translocations is hybrid (Wang et al. 2017). Structural rearrangements of compensating 2S^k^.2R centric translocations were observed in Sekundo-2S^k^.2R plants; however this was characteristic only for 18% of plants. Majority of Sekundo-2S^k^.2R plants (82%) revealed rejoined of 2S^k^ chromosome, which was puzzling (Fig. 3). It could be possible that compensating 2S^k^.2R Robertsonian translocations were broken and the meiotic cells with separated 2S^k^ chromosome arms that have rejoined into 2S^k^ and 2R chromosomes were functional. This assumption can be linked with the preferential transmission (Endo 2007) of 2S^k^ chromosome in wheat and triticale (Kwiatek et al. 2017) background, which is caused by gametocidal action.

Two groups of triticale translocation plants carried *Lr39* or *Lr54* leaf rust resistance genes were selected basing on cyto-molecular analysis. Both groups of plants were tested using a mixture of *P. triticina* races, which naturally occurred on the triticale plantations in Wielkopolska regions. Application of the natural mixture of the *P. triticina* pathotypes is the best way to evaluate the overall leaf rust resistance level and the usability of translocation plants of triticale for breeding purposes, as well. It is reported, that triticale is infected by the races specific to both: wheat and rye, however it was noticed that triticale is more easily attacked by the wheat physiological forms of the rusts than by the rye ones (Arseniuk 1996). It is also known that the rust species can hybridize through spontaneous crossings, e.g., *P. graminis* f. sp. *trifiei* and *P. graminis* f. sp. *secalis* on *Berberis vulgaris*, as well as somatically on graminaceous hosts observed in Sekundo-2S^k^.2R in comparison with triticale cv. Sekundo plants. The mean level of resistance was very high (1.59; 1.65 and 1.67 after 5, 10 and 15 days after infection, respectively). Such low infection rate can be considered as a result of *Lr54* gene expression. In similar study, Marais et al. (2005) developed a 2DS.2S^k^L wheat-*Ae. kotschyi* line (called S14 translocation) derived from the test cross of double monosomic 2D/*Ae. kotschyi* group2//_CS_-S) contained 96% resistant plants (72 tested) which were tested for resistance to eight *Pt* pathotypes (UVPrt2, UVPrt3, UVPrt4, UVPrt5, UVPrt8, UVPrt9, UVPrt10 and UVPrt13) and two *Pst* pathotypes (6E16A- and 6E22A-) endemic to South Africa. Moreover, it was reported that the S14 translocation evidently had preferential transmission (Marais et al. 2005). The results of inoculation test were similar to present study of 2R.2S^k^ translocation lines.

In comparison, the leaf rust resistance level did not differ between plants of Bogo-2D^t^.2R line and triticale cv. Bogo. What is more, the infection types were similar to those, scored on plants of KS90WGRC10 wheat line, which is reported to carry a *Lr39* leaf rust gene (Raupp et al. 2001; Gill et al. 2008). The parental forms for development Bogo-2D^t^.2R translocation line were selected from the monosomic 2D^t^ addition triticale genotypes carrying *Lr39* locus (Kwiatek et al. 2015; Majka et al. 2018). The leaf rust resistance of these genotypes was then determined at the macroscopic and microscopic level at the seedling (Majka et al. 2018). A board spectrum of *P. triticiana* was used including isolates virulent to *Lr39*. The results showed that hybrid plants revealed a limited level of leaf rust resistance at the seedling stage (Majka et al. 2018). The infection rate of Bogo-2D^t^.2R plants was comparable with results of inoculation performed on plants of triticale cv. Bogo. Majka et al. (2018) tested this cultivar, as well as monosomic addition (M2D^t^) lines of cv. Bogo, using a board spectrum of pure leaf rust isolates, which showed diverse response of Thatcher NILs containing *Lr39* gene (including complete and limited virulence). It was reported that triticale cv. Bogo is already very resistant and the introgression of complete 2D^t^ chromosome with *Lr39* gene showed no additional effect (Majka et al. 2018), which is a similar to the results obtained in present study of Bogo-2D^t^.2R translocation plants.

In summary, it could be said that only *Lr54* gene provided a significant improvement of the leaf rust resistance of triticale cv. Sekundo. Considering the lack of knowledge about the mechanisms of *Lr54* gene expression, the next step for this research appears to be the evaluation of *Lr54* gene transcription activity in triticale genetic background. Taking into consideration the application possibilities, these genetic stocks seem to be promising plant materials in the context of triticale resistance breeding. Moreover, the perspective of *Lr39* and *Lr54* genes pyramidisation is taken into account. However, further investigation aiming in the evaluation of the linkage drag effect on the yield, plant morphology and quality traits is required.

## Supplementary Information

Below is the link to the electronic supplementary material.

Supplementary file1 (DOCX 16 KB)

Supplementary file2 (DOCX 17 KB)

Supplementary file3 (DOCX 16 KB)

## Data Availability

The data that support the findings of this study are available from the corresponding author upon reasonable request.
