# Supplementary material for: Molecular identification of triticale introgression lines carrying leaf rust resistance genes transferred from Aegilops kotschyi Boiss. and Ae. tauschii Coss
Source: J Appl Genet. 2021 May 14;62(3):431–9. doi: 10.1007/s13353-021-00635-2 (PMC8357765; doi:10.1007/s13353-021-00635-2)
Supplement: Supplementary file 1 — Supplementary file1 (DOCX 16 KB) [file 13353_2021_635_MOESM1_ESM.docx]

**Supporting Information 1.** Analysis of variance (ANOVA) and Tukey’s HSD test for leaf rust infection scores (independent samples) of three groups of plants: 1) triticale cv. Bogo, 2) triticale cv. Sekundo, 3) wheat cv. Michgan Amber (infection control) and 4) KS90WGRC10 (*Lr39*). HSD - the absolute (unsigned) difference between any two sample means required for significance at the designated level HSD[.05] for the .05 level; HSD[.01] for the .01 level.

| ***Data Summary*** | | | | | | |
| --- | --- | --- | --- | --- | --- | --- |
|  | Groups | | | | Total | |
|  | 1 | 2 | 3 | 4 |  |  |
| Numer of plants | 30 | 30 | 30 | 30 | 120 | |
| Σ | 122 | 215 | 264 | 124 | 725 | |
| Mean | 4.0667 | 7.1667 | 8.8 | 4.1333 | 6.0417 | |
| ΣX^2^ | 504 | 1553 | 2328 | 520 | 4905 | |
| Variance | 0.2713 | 0.4195 | 0.1655 | 0.2575 | 4.41 | |
| Std. Dev. | 0.5208 | 0.6477 | 0.4068 | 0.5074 | 2.1 | |
| Std. Error | 0.0951 | 0.1183 | 0.0743 | 0.0926 | 0.1917 | |
| ***ANOVA summary*** | | | | | | |
| Source | SS | df | MS | F | | P |
| Treatment  (between groups) | 439.4917 | 3 | 164.1639 | 589.57 | | <.0001 |
| Error | 32.3 | 116 | 0.0284 |  |  | |
| Total | 524.7917 | 119 |  |  |  | |
| ***Tukey HSD test:* HSD_0.05_ = 0.33; HSD_0.01_ = 0.41** | | | |  |  |  |
| Mean of group 1 vs mean of group 2 | | | P<.01 |  |  |  |
| Mean of group 1 vs mean of group 3 | | | P<.01 |  |  |  |
| Mean of group 1 vs mean of group 4 | | | nonsignificant |  |  |  |
| Mean of group 2 vs mean of group 3 | | | P<.01 |  |  |  |
| Mean of group 3 vs mean of group 4 | | | P<.01 |  |  |  |
