# Supplementary material for: Molecular identification of triticale introgression lines carrying leaf rust resistance genes transferred from Aegilops kotschyi Boiss. and Ae. tauschii Coss
Source: J Appl Genet. 2021 May 14;62(3):431–9. doi: 10.1007/s13353-021-00635-2 (PMC8357765; doi:10.1007/s13353-021-00635-2)
Supplement: Supplementary file 2 — Supplementary file2 (DOCX 17 KB) [file 13353_2021_635_MOESM2_ESM.docx]

**Supporting Information 2.** Analysis of variance (ANOVA) and Tukey’s HSD test for leaf rust infection scores (independent samples) of Bogo-2D^t^.2R translocation line after: 1) 5; 2) 10; 3) 15 days post inoculation (dpi); 4) cv. Bogo and 5) KS90WGRC10 wheat line. HSD - the absolute (unsigned) difference between any two sample means required for significance at the designated level HSD[.05] for the .05 level; HSD[.01] for the .01 level. M – mean for sample.

| ***Data Summary*** | | | | | | |
| --- | --- | --- | --- | --- | --- | --- |
|  | Bogo-2D^t^.2R translocation line | | | cv. Bogo | KS90WGRC10 wheat line | Total |
|  | *days post infection (dpi)* | | |  |  |  |
|  | *5* | *10* | *15* |  |  |  |
| **Sample no.** | **1** | **2** | **3** | **4** | **5** |  |
| Numer of plants | 100 | 100 | 100 | 30 | 30 | 360 |
| Σ | 394 | 413 | 420 | 122 | 124 | 1473 |
| Mean | 3.94 | 4.13 | 4.2 | 4.0667 | 4.1333 | 4.0917 |
| ΣX^2^ | 1594 | 1737 | 1804 | 504 | 520 | 6159 |
| Variance | 0.4206 | 0.3163 | 0.404 | 0.2713 | 0.2575 | 0.3676 |
| Std. Dev. | 0.6485 | 0.5624 | 0.6356 | 0.5208 | 0.5074 | 0.6063 |
| Std. Error | 0.0649 | 0.0562 | 0.0636 | 0.0951 | 0.0926 | 0.032 |
| ***ANOVA summary*** | | | | | | |
| **Source** | **SS** | **df** | **MS** | **F** | **P** |  |
| Treatment  (between groups) | 3.6917 | 4 | 0.9229 | 2.55 | 0.039029 |  |
| Error | 128.2833 | 355 | 0.3614 |  |  |  |
| Total | 131.975 | 359 |  |  |  |  |
| ***Tukey HSD test:* HSD_0.05_ = 0.32; HSD_0.01_ = 0.39** | | | |  |  |  |
| M1 vs M2 | | | nonsignificant |  |  |  |
| M1 vs M3 | | | nonsignificant |  |  |  |
| M1 vs M4 | | | nonsignificant |  |  |  |
| M1 vs M5 | | | nonsignificant |  |  |  |
| M2 vs M3 | | | nonsignificant |  |  |  |
| M2 vs M4 | | | nonsignificant |  |  |  |
| M2 vs M5 | | | nonsignificant |  |  |  |
| M3 vs M4 | | | nonsignificant |  |  |  |
| M3 vs M5 | | | nonsignificant |  |  |  |
| M4 vs M5 | | | nonsignificant |  |  |  |
