# Supplementary material for: Molecular identification of triticale introgression lines carrying leaf rust resistance genes transferred from Aegilops kotschyi Boiss. and Ae. tauschii Coss
Source: J Appl Genet. 2021 May 14;62(3):431–9. doi: 10.1007/s13353-021-00635-2 (PMC8357765; doi:10.1007/s13353-021-00635-2)
Supplement: Supplementary file 3 — Supplementary file3 (DOCX 16 KB) [file 13353_2021_635_MOESM3_ESM.docx]

**Supporting Information 3.** Analysis of variance (ANOVA) for leaf rust infection scores (independent samples) of Sekundo-2S^k^.2R translocation line after: 1) 5, 2) 10 and 3) 15 days post inoculation (dpi) and 4) cv. Sekundo. HSD - the absolute (unsigned) difference between any two sample means required for significance at the designated level HSD[.05] for the .05 level; HSD[.01] for the .01 level. M – mean for sample.

| ***Data Summary*** | | | | | |
| --- | --- | --- | --- | --- | --- |
|  | Sekundo-2S^k^.2R translocation line | | | cv. Sekundo | Total |
|  | *days post infection (dpi)* | | |  |  |
|  | *5* | *10* | *15* |  |  |
| **Sample no.** | **1** | **2** | **3** | **4** |  |
| Numer of plants | 100 | 100 | 100 | 30 | 330 |
| Σ | 165 | 167 | 159 | 215 | 706 |
| Mean | 1.65 | 1.67 | 1.59 | 7.166667 | 2.139394 |
| ΣX^2^ | 307 | 311 | 277 | 1553 | 2448 |
| Variance | 0.35101 | 0.324343 | 0.244343 | 0.41954 | 2.849811 |
| Std. Dev. | 0.592461 | 0.569512 | 0.494311 | 0.647719 | 1.688138 |
| Std. Error | 0.059246 | 0.056951 | 0.049431 | 0.118257 | 0.092929 |
| ***ANOVA summary*** | | | | | |
| **Source** | **SS** | **df** | **MS** | **F** | **P** |
| Treatment  (between groups) | 834.371212 | 3 | 278.123737 | 878.43 | <.0001 |
| Error | 103.216667 | 326 | 0.316616 |  |  |
| Total | 937.587879 | 329 |  |  |  |
| ***Tukey HSD test:* HSD_0.05_ = 0.26; HSD_0.01_ = 0.31** | | | |  |  |
| M1 vs M2 | | | nonsignificant |  |  |
| M1 vs M3 | | | nonsignificant |  |  |
| M1 vs M4 | | | P<.01 |  |  |
| M2 vs M3 | | | nonsignificant |  |  |
| M2 vs M4 | | | P<.01 |  |  |
| M3 vs M4 | | | P<.01 |  |  |
